# Editorial: Progress in Computer Gaming and Esports: Neurocognitive and Motor Perspectives

**DOI:** 10.3389/fpsyg.2021.686152

**Published:** 2021-04-22

**Authors:** Adam J. Toth, Cornelia Frank, David Putrino, Mark J. Campbell

**Affiliations:** ^1^Department of Physical Education and Sport Sciences, Faculty of Education and Health Sciences, University of Limerick, Limerick, Ireland; ^2^Lero, The Irish Software Research Centre, Limerick, Ireland; ^3^Institute for Sport and Movement Sciences, University of Osnabrück, Osnabrück, Germany; ^4^Icahn School of Medicine, Mount Sinai Hospital, New York, NY, United States

**Keywords:** esport science, cognition, video games (psychology), AVG, sensory-motor abilities

This Research Topic covers the neurocognitive aspects of computer gaming and esports. Authors representing a broad spectrum of psychology and neuroscience have contributed, introducing empirical findings as well as conceptual and methodological innovations. In this Editorial we provide a thematic overview of the exciting and diverse contents of this Research Topic.

Video games have become a cultural phenomenon over the past 50 years and are now one of the most prominently chosen past times (Wagner, 2006; Hamari and Sjöblom, 2017; Lokhman et al., 2018). The use of dynamic visual displays, the demand on flexible attention allocation and the requirement for precise time-constrained bimanual motor control, make video games a unique medium for studying both cognition and motor control Bera et al. Over the past 20 years, neurocognitive research has demonstrated that habitual competitive video game players appear to display some superior cognitive attributes when compared to their non-video gaming counterparts (Colzato et al., 2013; Bediou et al., 2018; Kowal et al., 2018). Along with the increased recognition of esports as a sporting activity alongside traditional athletic sports, the unique cognitive skillset possessed by elite gamers has earned them the moniker of “cognitive athletes” (Campbell et al., 2018). This notion has led to an increased appetite toward understanding the cognitive benefits conferred from, and demanded by, video games.

A plethora of research has demonstrated that habitual action video gamers demonstrate superior information processing (Yuji, 1996; Dye et al., 2009; Kowal et al., 2018), attention Li et al.; Schenk et al., task switching (Colzato et al., 2010; Green et al., 2012; Toth et al., 2020), and memory abilities (Wilms et al., 2013; Waris et al., 2019), compared to non-gaming populations. Moreover, research has found these same cognitive skills can be enhanced when non-gamers engage with action video games in particular (Oei and Patterson, 2013; Clemenson and Stark, 2015). In fact, cognitive ability, in addition to past gaming experience, has even been found to predict one's ability to quickly learn new video games Smith et al. Alternatively, for some cognitive skills like response inhibition, gamers adopt a strategy that prioritizes speed over accuracy, showing no clear advantage (Kowal et al., 2018). This finding has been recently corroborated by Sousa et al., who demonstrated a decrease in inhibitory performance among participants following FPS and MOBA game play, which was larger in magnitude following FPS, compared to MOBA, play Sousa et al. Moreover, cognitive inhibition has also recently been shown not to differentiate ranking among gamers within a prominent FPS game (Colzato et al., 2013; Toth et al.). However, it is important to note that although classical tests of cognitive inhibition fail to differentiate gaming process, altering the context in which this skill is evaluated may also alter outcomes. For example, a gamified examination of cognitive inhibition may demonstrate performance differences among gamers, where classical standardized stroop tasks have failed to delineate differences. During F1 driving simulations for example, Eckardt et al. found that driving performance was largely tied to adaptive abilities and selective attention/inhibition ability Eckardt et al.

The recognized role of cognitive development and training in esports has extended to traditional sports, where superior executive function (EF) abilities can augment motor skill performance. For example, Beavan et al. share the importance of not only evaluating EF ability among soccer players, but also the set-up of consistent protocols and the effective communication of results to players. Traditional sport may also benefit from video games for motor learning too. In addition to the cognitive benefits that can be attained through video game play, video game environments have shown to promote motor skill practice away from the field of play. Michalski et al. demonstrate, in their recent review, that virtual environments (VE) now demonstrate a realism and flexibility that makes them potentially ideal and cost-effective for motor learning and the transfer of skill to the real world Michalski et al. Although work in this area is still sparse, early signs point to a promising role for VE to augment performance in traditional sports as well (Hülsmann et al., 2019).

In addition to the potential for video games to augment cognitive and motor abilities, they have also shown promise as a mental health tool as well (Kowal et al., 2021). Not only has this been significant in light of the recent COVID19 pandemic, but Tabacof et al. have demonstrated the critical role that video games can play in providing feelings of social connectedness among those who may have physical or mental disability Tabacof et al. In their study, they present solutions that can improve the accessibility of video games to those with spinal cord injury (SCI) and quadriplegia and how doing so significantly improves feelings of social connectedness among these individuals. Overall, this highlights the benefit that the digital nature of video gaming brings as a medium to remove barriers that may exist between individuals of varying sex, race, and physical ability.

Despite all the benefits to be had from video games, they are not without their pitfall. Their sedentary nature and the fact that users can find themselves staring at bright luminant screens for prolonged periods of time have been well cited as negative attributes (Lanningham-Foster et al., 2006; DiFrancisco-Donoghue et al., 2019; Yin et al., 2020). Therefore, some are now calling for a holistic approach to esports training and video game play in general Martin-Niedecken et al. In fact, new research is now altering the way esports players train, moving away from predominant full emersion training to adopting segmented skill based variable priority training strategies, prevalent in traditional sport (Toth et al., 2021). The role of physical exercise is also emerging as critical to the health and performance longevity in esports, despite physical exertion being a fraction of what is required at the elite level in traditional sports. Moreover, in addition to the well-established physical and mental benefits, physical exercise has been shown to augment the cognitive abilities which provide top video gamer players their advantage in the first place (Toth et al., 2020).

In conclusion, despite the prevalence and popularity of video games and esports worldwide today, we are still at the frontier of esports science and are only beginning to identify the key features that make elite esports athletes unique Smithies et al. Furthermore, the cognitive benefits conferred from engaging with video games, further social and physical benefits are likely to manifest as well, in addition to the development of a better understanding of how to mitigate health and performance pitfalls associated with video game play. As we embark on a new decade, we can expect to see a surge of new research on the role video games and virtual environments play in multiple facets of our lives, and this work is welcome now as we continue to place video games at the fore front of entertainment media in our digital world.

## Author Contributions

AT and MC wrote the first draft. CF and DP edited the draft. All authors contributed to and signed off on the final draft.

## Conflict of Interest

The authors declare that the research was conducted in the absence of any commercial or financial relationships that could be construed as a potential conflict of interest.
